# Synergy between Repellents and Organophosphates on Bed Nets: Efficacy and Behavioural Response of Natural Free-Flying *An. gambiae* Mosquitoes

**DOI:** 10.1371/journal.pone.0007896

**Published:** 2009-11-19

**Authors:** Cédric Pennetier, Carlo Costantini, Vincent Corbel, Séverine Licciardi, Roch K. Dabiré, Bruno Lapied, Fabrice Chandre, Jean-Marc Hougard

**Affiliations:** 1 UR016-CCPV (Caractérisation et Contrôle des Populations de Vecteurs), IRD (Institut de Recherche pour le Développement), Montpellier, France; 2 UR016-CCPV, IRD, Yaoundé, Cameroon; 3 UR016-CCPV, IRD, Cotonou, Benin; 4 UR016-CCPV, IRD, Saint-Denis, France; 5 IRSS (Institut de Recherche en Sciences de la Santé), Centre Muraz, Bobo-Dioulasso, Burkina-Faso; 6 RCIM, Université d'Angers, Angers, France; 7 UR016-CCPV, IRD, Dakar, Senegal; University of Swansea, United Kingdom

## Abstract

**Background:**

Chemicals are used on bed nets in order to prevent infected bites and to kill aggressive malaria vectors. Because pyrethroid resistance has become widespread in the main malaria vectors, research for alternative active ingredients becomes urgent. Mixing a repellent and a non-pyrethroid insecticide seemed to be a promising tool as mixtures in the laboratory showed the same features as pyrethroids.

**Methodology/Principal Findings:**

We present here the results of two trials run against free-flying *Anopheles gambiae* populations comparing the effects of two insect repellents (either DEET or KBR 3023, also known as icaridin) and an organophosphate insecticide at low-doses (pirimiphos-methyl, PM) used alone and in combination on bed nets. We showed that mixtures of PM and the repellents induced higher exophily, blood feeding inhibition and mortality among wild susceptible and resistant malaria vectors than compounds used alone. Nevertheless the synergistic interactions are only involved in the high mortality induced by the two mixtures.

**Conclusion:**

These field trials argue in favour of the strategy of mixing repellent and organophosphate on bed nets to better control resistant malaria vectors.

## Introduction

Malaria control aims to reduce or to interrupt transmission, either by attacking the parasite in the human host, or by attacking the mosquito vector at its various stages. Usually a combination of methods, integrated to suit local conditions, needs and available resources, is the most effective, but also the most difficult to apply. Malaria parasites are now extensively resistant to the majority of cheap and easy to use anti-malarial drugs [1]. The problem of drug resistance and the absence of a malaria vaccine available for use in the tropics in the near future, call for increased emphasis on vector control strategies in the control of malaria [2], [3]. To efficiently control plasmodium transmission by vectors, 1) the mosquito vector and its host-seeking behaviour must be well characterised and 2) the impact on the vector behaviour of vector control strategies and chemicals must be well studied.

In Western Africa, the major vector of malaria is *Anopheles gambiae* Giles *sensu stricto*, which is known to be anthropophilic, endophagic and endophilic [4], [5]. These characteristics are part of the reason that Insecticide Treated Nets (ITNs) are the mainstays of malaria vector control in these countries. Pyrethroids are recommended by the World Health Organization for bed net impregnation because they are effective at low dosages, fast acting, irritant and safe for humans [6]. Unfortunately pyrethroid resistance is widespread throughout Africa, especially with the target site mutation known as Knock down resistance (*Kdr*) [7], [8], [9], [10], [11]. Resistance mechanisms (i.e. *Kdr* and metabolic resistance mechanisms) might threaten sustainable vector control programs based on ITNs [12].

Recently, a new concept has been proposed: mixing a repellent and a non-pyrethroid insecticide on a net. Such mixtures showed similar features of pyrethroids, i.e. the lethal effect, knock-down effect and irritancy against susceptible and pyrethroid-resistant mosquitoes [13], [14]. Two combinations (using pirimiphos methyl (PM), an organophosphate, and two repellents, diethyl-m-toluamide (DEET) and KBR 3023 also known as icaridin) were chosen to be tested in the field. Pennetier *et al.* [15]found that the bed nets treated with the two mixtures were as effective as deltamethrin against susceptible mosquitoes, and more effective in killing *Anopheles gambiae* carrying *Kdr* or *Ace.1^R^* resistance genes. Moreover the mixtures did not select for either the *Kdr* or the *Ace.1^R^* alleles indicating that Repellent/Insecticide Treated Nets (RITNs) could be used to control insecticide-resistant malaria vectors[15].

The key factors in this promising strategy are quite volatile compounds, the repellents. As emphasized by Grieco *et al.* [16], chemicals cannot be classified based solely on their killing effect. They can disrupt contact between humans and malaria-transmitting mosquitoes not only by killing the mosquitoes. Indeed, the first host cues to reach a mosquito are (after carbon dioxide) volatile chemicals emanating from the skin, breath and waste products of a potential host, carried by air currents[17]. The probability that the mosquito responds to these cues and the strength of the response depend on the strength of the host-derived stimuli, the strength of competing external stimuli (e.g. odours from other sources, prohibitive wind speeds, etc.), the internal state of the mosquito (e.g. circadian phase, gonotrophic status, etc.) and its genotype (i.e. the genetic component of the responsiveness to given stimuli)[18]. ITNs constitute external stimuli sources because of chemicals on it, which are also released in their vicinity. *An. gambiae* behaviour in response to both human-derived stimuli and ITN stimuli is unknown, as is the behaviour in response to a physical barrier (untreated bed nets).

Usually, studies about ITNs efficacy evaluate the following parameters: deterrence, induced exophily, blood feeding inhibition and induced mortality. Using these indicators, we aimed to better understand the behavioural modifications and insecticidal efficacy induced by bed nets impregnated with PM/repellent mixtures and to investigate the involvement of the positive interactions between active compounds using experimental huts [19]. To do this, we compared the efficacies of the two mixtures with the compounds used alone on mosquito nets relative to an untreated net against free-flying malaria vectors in Burkina Faso, West Africa.

## Methods

### Study Area

Vallée de Kou (11°24′N; 04°24′W) is about 30 km north-west of Bobo-Dioulasso in the valley of the Kou River, a region where there has been extensive rice cultivation since the 1970s. This area contains 7 villages covering 7,200 ha surrounded by wooded savannah. As the Kou River flows all year round, it offers a permanent source of water for irrigation, hence allowing two crops of rice per year (July-November and January-May). The rice fields are highly productive permanent mosquito breeding sites, especially for the molecular form M of *An. gambiae*. In rainy season additional anopheline breeding sites appear, such as depressions and rain puddles, where the molecular form S is more prevalent.

### Repellents and Insecticides

An organophosphate insecticide and two repellent products were evaluated separatly and mixed on nets: “Pirigrain 250”, a product produced by the company CGI (Compagnie Générale des Insecticides, France), is a formulation containing 25% Pyrimiphos methyl (PM); “DEET” is a formulation containing 30% diethyl-3-methylbenzamide (Deet); and “Icaridin®” or “Bayrepel®” is a formulation containing 25% KBR 3023, both produced by the company Osler, France. All three products were liquid (EC) formulations designed for clothing application. No other toxic or repellent chemicals other than those mentioned above were declared in the formulations.

### Nets and Impregnation

Six polyester nets of 100 denier netting and 156 mesh size were used in the study. To simulate the usually reported torn nets usually reported, 6 holes each measuring 4 cm × 4 cm were cut in the sides and ends of the nets according to WHO guidelines [19]. The nets were impregnated before each trial with 150 mg/m^2^ PM and 10 g/m^2^ of both DEET and KBR, alone or in combination.

### Experimental Huts and Mosquito Collections

Experimental hut procedures and mosquito collections were carried out as performed by Darriet *et al*. [20]. Briefly, adult male volunteers slept in the huts on mats under the nets from 20:00 to 05:00 each night after cleaning the hut at 18:00 to remove any spiders or other predators. To minimise bias in individual attractiveness, sleepers and bed nets were rotated between huts on successive nights following a Latin square. Awaking at 05:00, the sleeper closed the windows, lowered the curtain separating the room from the verandah, and collected live and dead mosquitoes from the room, bed net, and veranda. Female mosquitoes were scored as dead or alive, fed or unfed, and identified to species. Two trials,one in the dry season (May 5^th^ to June 3^rd^) and one in the wet season September 18^th^ to October 14^th^, 2006) lasted each 27 nights.

The entomological impact of each treatment on mosquitoes was expressed relative to the control in terms of:

- Deterrency: the reduction in the number of mosquitoes found in a treated hut.
- Exophily: the proportion of mosquitoes found in the veranda of a treated hut.
- Blood feeding rate: the proportion of mosquitoes caught that were blood fed.
- Overall mortality rate: the proportion of mosquitoes found dead immediately (at time of collection) and after 24 h to account for delayed mortality.

### PCR Detection of Resistance Alleles

Genomic DNA was extracted from field-collected mosquitoes and PCR amplified to determine the presence of the molecular forms M or S using the method of Favia et al.[21]. Samples of live and dead mosquitoes were taken from the control hut for detection of *kdr* and *Ace1^R^* alleles in individual mosquitoes using respectively the methods of Martinez-Torrez et al. [7] and Weill et al. [22].

### Statistical Analysis

The response variable y was the number of dead mosquitoes each day. The fraction of dead mosquitoes p  =  y/n (where n is the total number of mosquitoes collected in the hut) was related to the time, the treatment of the bed net, the blood feeding rate b  =  bfd/n, the exophily q  =  exo/n and the season in a logistic regression model with the software GLIM v.4 [23]. The model assumed a binomial error distribution with regression parameters calculated by maximum likelihood. The statistical significance of main effects and interactions terms in the model was tested by F-tests in an analysis of the deviance (anodev) by looking at the change in deviance caused by the removal of each term from the maximal model after having allowed for over dispersion in the data by calculating heterogeneity coefficients with the Williams algorithm [24], [25]. Exophily and blood feeding rates were analysed following the same maximum likelihood procedures in GLIM v.4 software. Numbers of mosquitoes entered in the huts were related to time (days) in a model assuming a poisson error distribution with regression parameters also calculated by maximum likelihood using GLIM v.4 software.

### Ethical Considerations

Volunteers from the study village were recruited after obtaining informed written consent. A medical doctor was on hand during the trial to respond to any side effects of the ITNs or to treat any cases of fever. Confirmed *falciparum* parasitaemia was treated with Coartem (artemether 20 mg/lumefantrine 120 mg). The protocol received approval from the ethics committees of Centre Muraz (a national research centre) and Institut de Recherche pour le Developpement.

## Results

### Vector Population Composition and Insecticide Resistance Status

A total of 6932 *An. gambiae sl* has been collected among the two trials (3768 *An. gambiae* between May 8^th^ and June 3^rd^ 2006; 3164 *An. gambiae* between September 17^th^ and October 13^th^ 2006). Two sub-samples of among 50 mosquitoes from the control hut of each trial were molecularly characterized for the molecular form and the resistance status. Molecular analysis revealed, as expected, a marked change between seasons in molecular form composition and insecticide resistance status (Table 1). During the dry season the molecular form S accounted for 5% of the *Anopheles gambiae s.s.* population, whereas at the end of the dry season it represented 85% of the sample. Accordingly, the *Kdr* allele, which confers resistance to pyrethroids, was found at a frequency of 8% in the *An. gambiae* s.s. sample during the dry season replicate and at 88% at the end of the rainy season. Similarly, the frequency of the *Ace1^R^* allele, which confers resistance to organophosphates and carbamates, increased from 1% at the end of the dry season to 40% during the rainy season. The change in frequency of the insecticide resistance genes reflects the fact that these genes are found at high frequency only in the molecular form S of *An. gambiae*.

**Table 1:** Frequency of the S molecular form of *An. gambiae*, of Knock Down Resistance (*Kdr*) allele and insensitive acetylcholinesterase (*Ace.1^R^*) allele.

	S form frequency (n tested)	*Kdr* frequency (n tested)	*Ace1^R^* frequency (n tested)
dry season	0,05 (43)	0,08 (41)	0,01 (40)
rainy season	0,85 (49)	0,88 (48)	0,40 (49)

Samples have been randomly taken in the control hut.

### Deterrency

**Figure 1:**
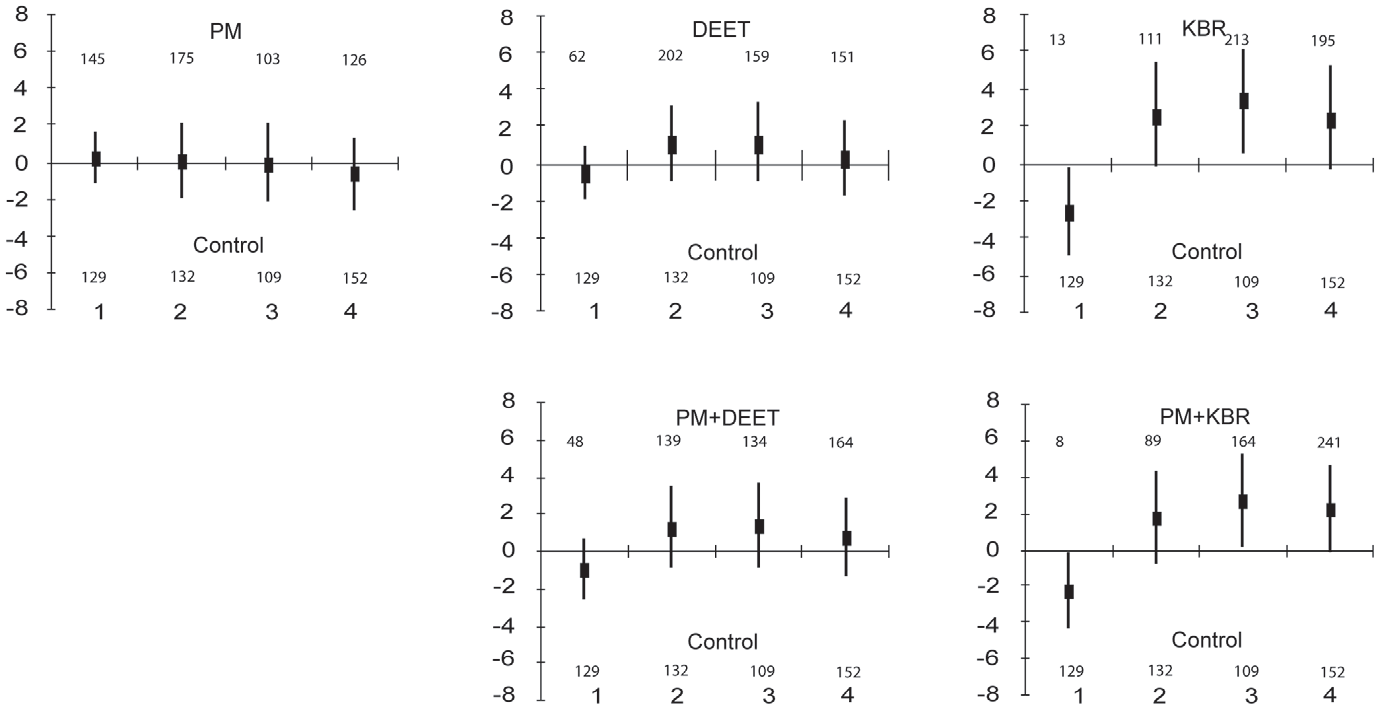
Log of the difference between numbers of mosquitoes entering treated hut and the control one, with 95% confidence intervals. Axis of abscissa represents the week numbers after the beginning of the trial. For each week are written the number of *An. gambiae* mosquitoes collected in the treated hut (at the top) and the number of *An. gambiae* mosquitoes collected in the control hut.

Analysis of the variance of the number of *An. gambiae* caught in huts with different treatments indicated that the only statistically lower density in treated huts occurred during the first week. Entry rates were significantly lower from the control for both KBR and PM+KBR treatments (95% confidence intervals did not overlap 0) (fig. 1c & 1e). PM, DEET and PM+DEET did not induce any differences from the control in terms of the *An. gambiae* entry rate during the 4 weeks (95% CI overlapped 0) (fig. 1a, 1b, 1d). Note that significantly more mosquitoes entered in huts where there were KBR and PM+KBR treated nets during the 3^rd^ week, indicating an attractive effect of these bed nets (fig. 1c & 1e).

### Exophily

**Figure 2:**
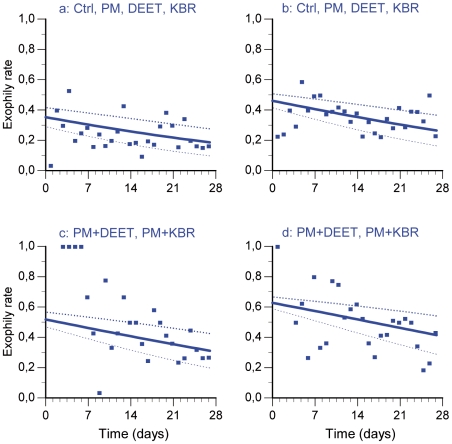
Exophily rates over time in the minimal adequate logistic regression model with standard error bounds. a and c illustrate the trial during dry season 2006; b and d illustrate the trial during rainy season 2006.

In the minimal adequate model for the exophily data, there was neither difference between the control, PM and the two repellents used alone nor between the two mixtures. Data of control, PM, DEET and KBR treatments have therefore been pooled (fig. 2a & 2b) as well as the data of the two mixtures PM+DEET and PM+KBR (fig. 2c & 2d). Exophily induced by the two mixtures (fig. 2c) was higher than the control and compounds used alone (fig. 2a) (t = 6.83; p<0.005). The same trend was observed in rainy season but populations of *An. gambiae* seemed to be more exophilic than in dry season (t = 4.71; p<0.005) (fig. 2b & 2d).

The involvement of the interactions in the exophily rate was tested in another model in which we replaced the factor “treatment” by two factors “repellent” and “insecticide”. This model allowed us to show that the interactions between PM and the two repellents are not involved in the increase of expophily (F = 1.52; p = 0.22). In others words, the increased exiting behaviour induced by the mixture treatments are not due to synergistic interactions between PM and the two repellents DEET and KBR but to additive effect of the compounds.

### Blood Feeding

**Figure 3:**
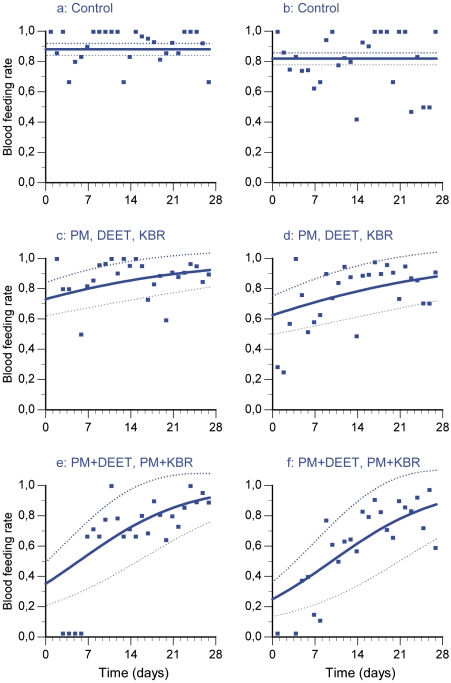
Blood feeding rates over time in the minimal adequate logistic regression model standard error bounds. a, c and e illustrate the trial during dry season 2006; b, d and f illustrate the trial during rainy season 2006.

Blood feeding rates were constant within each trial in the control huts (fig. 3a & 3b). In dry season, it was higher (88.0%±2.0) than in rainy season (81.9%±2.0) (t = 3.73; p<0.05). In the minimal adequate model for the blood feeding data, there is no difference neither between PM and the two repellents used alone nor between the 2 mixtures. Data of PM, DEET and KBR treatments have therefore been pooled (fig. 3c & 3d) as well as the data of the two mixtures PM+DEET and PM+KBR (fig. 3e & 3f). However, the mixtures inhibited significantly more blood feeding than the repellent compounds used alone (t = 7.70; p<0.005). The compounds used alone inhibited only 17% and 24% of the blood feeding respectively during the dry and rainy season (fig. 3c & 3d). The two mixtures inhibited 60% and 70% of the blood feeding respectively during the dry and rainy season (fig. 3e & 3f).

The involvement of the interactions in the blood feeding inhibition was tested in another model in which we replaced the factor “treatment” by two factors “repellent” and “insecticide”. This model allowed us to show that the interactions between PM and the two repellents are not involved in the decrease of blood feeding rate (F = 1.01; p = 0.36). In others words, the decreased blood feeding behaviour are not due to synergistic interactions between PM and the two repellents DEET and KBR but to additive effect of the compounds.

### Mortality

**Figure 4:**
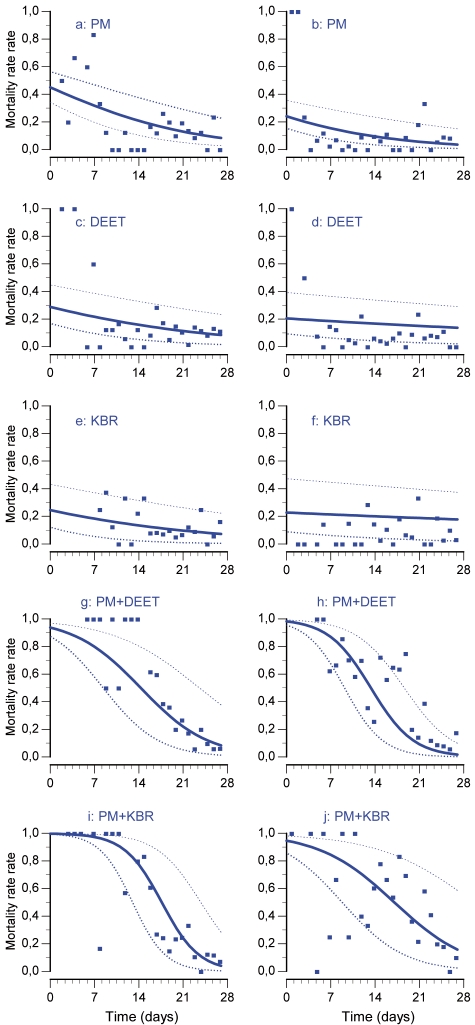
Mortality rates over time in the minimal adequate logistic regression model with standard error bounds. a, c, e, g and i illustrate the trial during dry season 2006; b, d, f, h and j illustrate the trial during rainy season 2006.

The model that best fit the data took into account the main effects treatment and time and their interaction with the season. Exophily and blood feeding explain a significant part of the deviance of the mortality data depending on the treatment (respectively f = 17.05; p = 0.043 and f = 16.59; p = 0.047). At the beginning of the dry season trial, PM was killing less than 50% of exposed mosquitoes (fig. 4a). DEET and KBR were killing less than 30% (fig. 4c & 4e). In contrast at the same time, PM+DEET was killing 93% of mosquitoes that entered in the hut (fig. 4g) and PM+KBR about 99% (fig. 4i).

In the rainy season, the mortality at the beginning of the trial was significantly lower than in the dry season for PM, DEET and KBR used alone (fig. 4b, 4d & 4f). The mortality induced by PM+DEET did not decrease significantly in the rainy season (t = 0.98; p>0.05) (fig. 4h), in contrast with PM+KBR (t = 4.32; p<0.005) (fig. 4j). Moreover the maximal efficacy did not last as long as it did in dry season (fig. 4h & 4j).

The involvement of the interactions in the blood feeding inhibition was tested in another model in which we replace the factor “treatment” by two factors “repellent” and “insecticide”. This model allowed us to show evidence of synergistic interactions between PM and the two repellents are involved in the mortality induced (F = 4,15; p = 0,016). The differences observed between the mixtures and compounds used alone are characteristic of their interactions. Positive interactions were greater between PM and KBR than between PM and DEET. Synergy amplitude was affected by the season change for PM+KBR (t = 4.32; p<0.01) but not for PM+DEET (t = 0.97; p>0.05). All the mortality estimates are summarized in the table 2. The mortalities induced by the two mixtures are much greater than the expected ones under the hypothesis of independent actions of the two compounds.

**Table 2:** Mortality estimates (Standard Error SE) from the GLM model and expected mortalities under the hypothesis of independent action induced by all treatments in the huts during the dry and rainy seasons.

	Dry season		rainy season	
	Mortality	SE	Mortality	SE
Control	5,77%	9,49%	2,47%	3,42%
PM	45,13%	15,17%	24,22%	24,21%
DEET	29,16%	15,38%	20,34%	19,95%
KBR	25,12%	18,54%	22,01%	15,66%
**Expected PM+DEET**	**61,13%**		**39,63%**	
PM+DEET	93,67%	9,54%	97,67%	12,43%
**Expected PM+KBR**	**58,92%**		**40,90%**	
PM+KBR	99,74%	0,64%	94,07%	30,49%

## Discussion

Many field studies have been run with insecticide mixtures for which synergistic interactions have been observed in laboratory [26], [27], [28]. But none of these showed evidence of synergistic interactions in field conditions. Our results showed for the first time synergism in natural conditions against wild populations of the main malaria vector, *An. gambiae*. This synergy occurred between PM and the two repellents DEET and KBR, as previously demonstrated [14]. Moreover, the two mixtures PM+DEET and PM+KBR were still efficient against *An. gambiae* populations that shared the *Ace1^R^* and *Kdr* resistance genes at high levels (*Ace1^R^* freq  = 0.40; *Kdr* freq  = 0.88). A companion study showed that these two mixtures are more efficient than conventional pyrethroid-treated nets against susceptible and resistant *An. gambiae* populations and did not induce any additional selective pressure on the resistance genes, *Ace1^R^* and *Kdr* [15]. Criticisms on this new strategy of resistant malaria vector control focused on the short residual effect of the repellents [29]. However, companies are now working to develop long-lasting repellent formulations. For example, a micro-encapsulated formulation (MC) of DEET showed residual efficacy for up to six months in laboratory conditions [30], [31]. Moreover, other DEET formulations are currently being evaluated in laboratory conditions and are showing the same efficiency one year after application one nets (Pennetier *et al*, unpublished data). So, it is not unrealistic to imagine that long-lasting repellent formulations will be available in the next few years.

The major factor preventing the immediate application of this kind of mixture on bed nets in the field is the lack of knowledge of the toxic properties of repellent-plus-OP mixtures. Indeed we used an OP with DEET which also acts as an acetylcholinesterase inhibitor [32] or with the KBR for which the mode of action is unknown. Despite the fact that these 2 repellents and PM are reported as safe products [33], [34], [35], [36], [37], little is known about the interaction of repellents with OPs. Moreover none of our compounds was applied on the skin. The contact between the user and the active ingredients on the bed net surface would be limited compared with a skin application, and the repellent concentration we used on nets was >3-fold lower than that recommended for a skin application (30% of DEET active ingredient in commercial lotions). Nevertheless, because a mixture of chemicals must be considered as a new chemical, assessing the risk of using repellent plus OP at the operational doses used to impregnate bed nets is crucial.

In the present study, blood feeding and exophily behaviour explained a part of the variability of the mortality of *An. gambiae* for the compounds when used alone, but not for the mixtures, indicating that the efficacy of the mixtures was not dependent on the mosquito behaviour in the experimental huts. Nevertheless the question of mosquito host-seeking behaviour in the presence of a physical barrier (bed net) or chemical (repellent on skin, ITNs, or volatile compounds in dwellings) is consequently crucial. Many fundamental studies have focussed on free host seeking behaviour (i.e. without any chemical or control tool) but there is a lack of knowledge about the behavioural accommodations of mosquitoes in the presence of treated materials as has been done for behaviour responses of Tsetse flies (*Glossina ssp*) to a vector control tool like insecticide treated cattle [38], in order to improve the control strategies of human african trypanosomiasis [39].

Here, the objective was to better understand the impact of interactions between PM and the two repellents, DEET and KBR 3023 so we used quite low dosages. It would be interesting to study behavioural modifications and the insecticidal effect of PM and the two repellents at higher dosages to investigate the potentialities of using these compounds alone on bed nets [40]. Investigations on repellents are all the more important as we showed that the same chemical (KBR 3023) could be repellent or attractant according to its concentration, as has already been demonstrated for DEET [41].

Nevertheless, chemicals are only external stimuli added to human cues. Our results also showed a significant effect of the season on different indicators, *An. gambiae* populations in the rainy season were significantly more exophilic and significantly less aggressive than in the dry season. There are too many differences between these two populations (genetic background, meteorological conditions) to hypothesize about the cause. But this indicates that there might be differences in host-seeking behaviour between the M and S molecular forms, between mosquitoes that are sharing different insecticide resistance genes, between mosquitoes with different parasitic status, or with different ecological or meteorological preferences. This information can lead us to improve the protocol of experimental hut trials, especially by including more variables (for example, parasitic status or meteorological data), and using a general model to take in account the impact of all these variables on the efficacy of different treatments. Standard protocols and new classifications proposed by Grieco *et al.* [16] should be the first step toward the establishment of general methods to evaluate new chemical proposed for malaria vector control.

In conclusion, our results showed a potential tool to manage resistant *An. gambiae*. Mixing OPs and repellents offered excito-repellency and mortality as required for protecting the sleeper and the community based on the positive interaction of the 2 chemicals. This concept will have practical potentialities for malaria control when long-lasting formulations of repellent are available. Generally, this study focused on the need to improve knowledge about mosquito vector host-seeking behaviour, particularly when treated materials are used.
